# Mixed Large Cell Neuroendocrine Carcinoma and Adenocarcinoma with Spindle Cell and Clear Cell Features in the Extrahepatic Bile Duct

**DOI:** 10.1155/2014/347949

**Published:** 2014-04-01

**Authors:** John Wysocki, Rishi Agarwal, Laura Bratton, Jeremy Nguyen, Mandy Crause Weidenhaft, Nathan Shores, Hillary Z. Kimbrell

**Affiliations:** ^1^Department of Gastroenterology and Hepatology, Tulane University, 1430 Tulane Avenue, New Orleans, LA 70112, USA; ^2^Department of Pathology and Laboratory Medicine, Tulane University, 1430 Tulane Avenue, SL-79, New Orleans, LA 70112, USA; ^3^Department of Radiology, Tulane University, 1430 Tulane Avenue, New Orleans, LA 70112, USA

## Abstract

Mixed adenoneuroendocrine carcinomas, spindle cell carcinomas, and clear cell carcinomas are all rare tumors in the biliary tract. We present the first case, to our knowledge, of an extrahepatic bile duct carcinoma composed of all three types. A 65-year-old man with prior cholecystectomy presented with painless jaundice, vomiting, and weight loss. CA19-9 and alpha-fetoprotein (AFP) were elevated. Cholangioscopy revealed a friable mass extending from the middle of the common bile duct to the common hepatic duct. A bile duct excision was performed. Gross examination revealed a 3.6 cm intraluminal polypoid tumor. Microscopically, the tumor had foci of conventional adenocarcinoma (CK7-positive and CA19-9-postive) surrounded by malignant-appearing spindle cells that were positive for cytokeratins and vimentin. Additionally, there were separate areas of large cell neuroendocrine carcinoma (LCNEC). Foci of clear cell carcinoma merged into both the LCNEC and the adenocarcinoma. Tumor invaded through the bile duct wall with extensive perineural and vascular invasion. Circumferential margins were positive. The patient's poor performance status precluded adjuvant therapy and he died with recurrent and metastatic disease 5 months after surgery. This is consistent with the reported poor survival rates of biliary mixed adenoneuroendocrine carcinomas.

## 1. Case Report

A 65-year-old man with a remote history of cholecystectomy for benign disease presented with a two-week history of painless jaundice, nausea, vomiting, and an unintentional 40-pound weight loss. His physical exam was within normal limits; specifically he was afebrile and did not have abdominal tenderness. Initial labs included a markedly elevated CA19-9 (2396 U/mL, normal range 0–35 U/mL), mildly elevated alpha fetoprotein (10.1 ng/mL, normal range 0.5–8.0 ng/mL), and normal CEA (1.3 ng/mL, normal range 0–3.0 ng/mL for non-smokers). Initial abdominal ultrasound demonstrated diffuse dilatation of the intrahepatic and common hepatic bile ducts. The largest intrahepatic duct had a diameter of 1.8 cm. At the level of the hepatic hilum, the common duct had a maximum diameter of 2.7 cm and a portion of the duct was filled with complex echogenic material. A triple phase liver CT showed a 3.8 × 2.5 × 2.1 cm enhancing mass in the expected region of the intra- and extrahepatic bile duct. Endoscopic retrograde cholangiopancreatography showed a severe filling defect measuring 1.7 cm in the middle portion of the common bile duct with proximal and distal dilation. Cholangioscopy demonstrated a soft, friable tumor, extending from the mid-common bile duct to the common hepatic duct; the tumor was biopsied and brushed during this procedure, but the specimens contained only necrotic debris.

**Figure 1:**
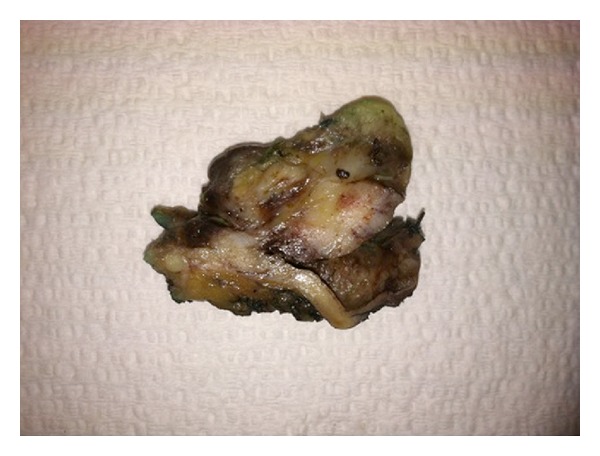


**Figure 2:**
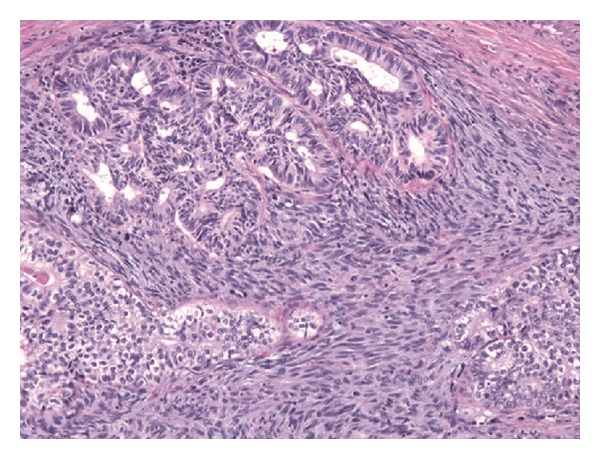


**Figure 3:**
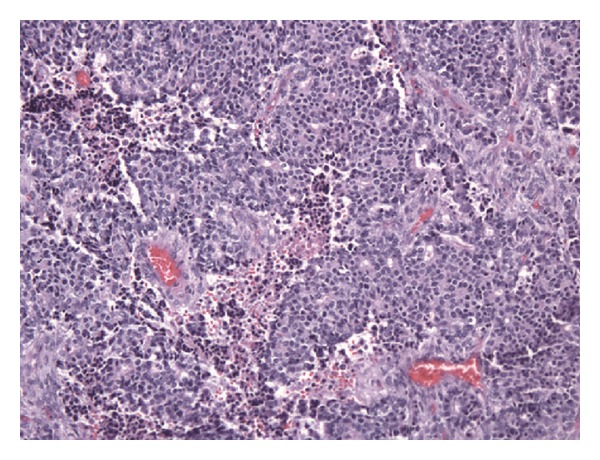


**Figure 4:**
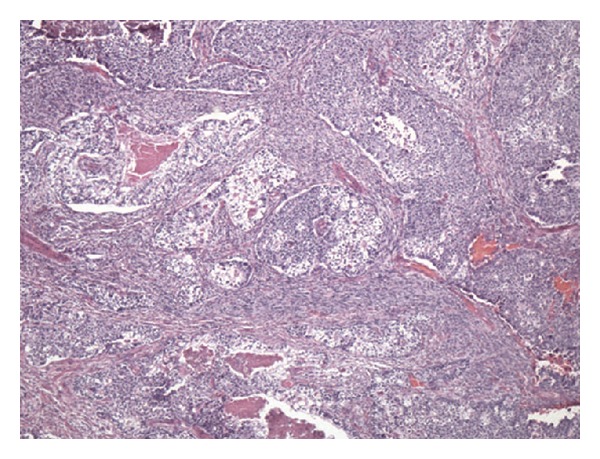


The patient was given a biliary stent and discharged with outpatient follow-up; however, he soon re-presented with worsening jaundice. Therefore, the patient underwent a bile duct excision with creation of a hepaticojejunostomy. A 5.0 × 3.5 × 2.8 cm segment of bile duct was removed. On gross examination, the bile duct contained a 3.6 cm intraluminal polypoid tumor (Figure 1). Microscopically, the tumor was composed of islands of conventional adenocarcinoma and clear cell carcinoma surrounded by malignant-appearing spindle cells (Figure 2). The spindle cells were positive for cytokeratins and vimentin, consistent with spindle cell carcinoma. Additionally, there were separate areas of large cell neuroendocrine carcinoma (LCNEC), which formed relatively broader sheets with focal rosette-like structures and abundant necrosis (Figure 3). Many tumor nests contained a mixture of LCNEC and clear cell carcinoma (Figure 4), and others contained mixtures of all three types. The tumor invaded through the wall of the bile duct into surrounding soft tissue. Lymphatic and vascular invasion were present, and tumor extended perineurally to the circumferential margins. Proximal and distal bile duct margins were negative. One lymph node was received and was negative for tumor.

**Figure 5:**
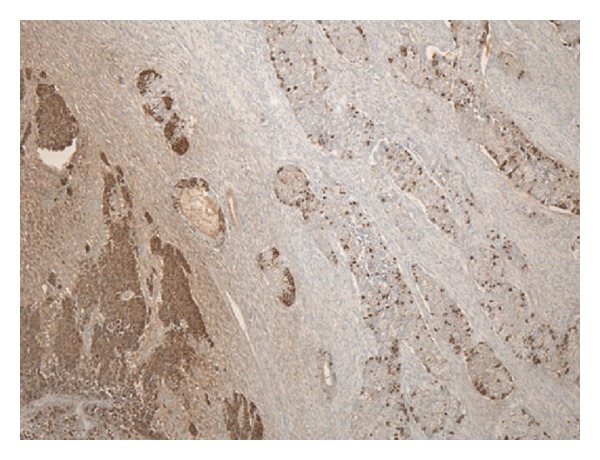


On immunohistochemistry, the neuroendocrine markers synaptophysin, chromogranin, and CD56 were positive in the LCNEC and in the adenocarcinoma, focally positive in the spindle cells, and showed scattered positive single cells in the clear cell carcinoma, mostly around the edges of the islands (Figure 5). AFP and HepPar 1 were expressed in the clear cell areas and also focally in the adenocarcinoma. CD117 was positive in the LCNEC only. All four components were positive for p53. The Ki-67 index was highest in the LCNEC and lowest in the clear cell carcinoma; interestingly, the Ki-67 tended to be positive in the periphery of the clear cell islands, which were the same cells that were positive for neuroendocrine markers.  Table 1 summarizes the immunohistochemical results.

The patient's performance status was too poor to receive any adjuvant therapy, and he died with recurrent and metastatic disease five months postoperatively.

## 2. Discussion

Our patient's tumor is a combination of LCNEC, spindle cell carcinoma, and clear cell carcinoma. To the best of our knowledge, this combination has not been previously reported. Each component in and of itself is rare in the extrahepatic bile duct and will be discussed individually.

It is difficult to determine the exact number of LCNEC reported in the biliary tract because of the evolving terminology for neuroendocrine neoplasms. In the tubular gastrointestinal (GI) tract, neuroendocrine neoplasms are divided into well-differentiated tumors (formerly known as carcinoid tumors) and high-grade carcinomas. High-grade neuroendocrine carcinomas can be further subdivided into small cell carcinomas and LCNEC based on morphologic features: small cell carcinomas are characterized by diffuse growth pattern, markedly high nuclear/cytoplasmic ratio, hyperchromatic nuclei with finely granular “salt and pepper” chromatin, inconspicuous nucleoli, and nuclear molding. In contrast, LCNECs have a “neuroendocrine architecture” of organoid or trabecular growth with nuclear palisading and/or rosette formation, relatively monotonous round-to-oval nuclei with more conspicuous nucleoli, moderate amounts of cytoplasm, and generally large foci of necrosis. Unlike small cell carcinoma, which is a morphologic diagnosis, LCNEC must demonstrate immunohistochemical staining for at least one neuroendocrine marker in >20% of the cells [1]. LCNECs in the tubular GI tract often have an associated adenomatous or adenocarcinomatous component [1]. Tumors composed of both adenocarcinoma and a neuroendocrine neoplasm are classified as “mixed adenoneuroendocrine carcinoma” (MANEC) in the 2010 WHO classification [2]. The WHO does not specify which type of neuroendocrine neoplasm should be present in a MANEC (i.e., well-differentiated versus high-grade). Although there are several reports of biliary MANECs with a small cell component, there have been only two previously reported cases of MANECs with a large cell neuroendocrine component in the extrahepatic bile duct [3, 4]. Presenting symptoms of biliary MANECs are nonspecific, including jaundice and abdominal pain. They tend to present at an advanced stage, and the metastases can be composed of adenocarcinoma, neuroendocrine carcinoma, or both [3, 5]. Although LCNEC on its own is a very aggressive tumor—in a small series of gallbladder LCNEC, patients showed rapid disease progression and poor response to systemic chemotherapy [6]—the prognosis of MANECs might be better than that of pure high-grade neuroendocrine carcinomas. In a series of nearly 5,000 gastroenteropancreatic and hepatobiliary neuroendocrine neoplasms in Korea, the 5-year survival rate for MANECs was 43% versus 35% for pure high-grade neuroendocrine carcinoma; this series included approximately 26 MANECs of the gallbladder and bile duct. Statistically significant prognostic indicators for MANECs in this study were lymph node status, tumor extension, and specific site of tumor, with small intestinal and pancreatic tumors having the best prognosis and esophageal the worst [7]. Similarly, in the tubular GI tract, patients with an adenocarcinoma component had a significantly better survival rate than patients with pure high-grade neuroendocrine carcinoma [1].

Similar to neuroendocrine neoplasms, spindle cell carcinoma has a confusing variety of names in the literature, such as pseudosarcoma and sarcomatoid carcinoma. By the 2010 WHO classification, spindle cell carcinoma falls under the heading of “undifferentiated carcinoma, spindle, and giant cell type” (UCSGT) [2]. UCSGT has several variants: spindle cell, giant cell, small cell (nonneuroendocrine) and nodular/lobular type. By definition, the tumor cells are positive for cytokeratins by immunohistochemistry and therefore are thought to be carcinomatous in nature. This is in contradistinction to carcinosarcomas, which also occur in the biliary tract [8]. Carcinosarcomas contain a true mesenchymal component (i.e., negative for cytokeratins), often in the form of heterologous elements such as chondrosarcoma, osteosarcoma, or rhabdomyosarcoma. UCSGT can also have areas of adenocarcinoma and squamous cell carcinoma [2, 9]. The prognosis for UCSGT is uncertain due to the rarity of cases.

There are only five reported cases of clear cell carcinoma in the extrahepatic bile duct [10–12]. Four of these five cases had foci of conventional adenocarcinoma in addition to the clear cell component. Our case produced and stained for alpha fetoprotein and also stained for HepPar 1, both of which have been reported previously [10, 11]. These features may cause confusion if hepatocellular carcinoma is in the differential diagnosis, especially since hepatocellular carcinoma can also undergo clear cell change. Since the clear cell carcinoma in our case was positive for CK7, it is unlikely to represent a component of clear cell hepatocellular carcinoma. It is not known if the prognosis of clear cell carcinoma is any different from conventional adenocarcinoma because there have been so few cases. Its recognition as an entity is important, however, so it is not confused with metastatic clear cell renal cell carcinoma. Clear cell carcinoma of the biliary tract should be positive for CK7 and negative for RCC and PAX8, whereas metastatic clear cell renal cell carcinoma would show the reverse pattern [2]. It should be mentioned that clear cell change has also been reported in a well-differentiated neuroendocrine tumor (carcinoid) of the distal common bile duct [13]. In our case, the clear cell component showed only scattered single cells that were positive for neuroendocrine markers and did not have an organoid growth pattern so therefore does not represent a clear cell variant of a neuroendocrine tumor.

There are only four other case reports of mixed tumors with multiple morphologies in the gastrointestinal and hepatobiliary tract: a tumor composed of small cell carcinoma, spindle cell carcinoma, adenocarcinoma, squamous cell carcinoma, and something the authors called “undifferentiated carcinoma” (small tumor cells with clear cytoplasm that were positive for neuroendocrine markers) in the gallbladder [9]; a mixed large cell neuroendocrine carcinoma and sarcomatoid carcinoma in the gastroesophageal junction [1]; a mixed hepatocellular carcinoma, spindle cell carcinoma and adenocarcinoma in the liver [14]; and a mixed neuroendocrine carcinoma, squamous cell carcinoma, and ciliated adenocarcinoma with spindle cell sarcoma in the esophagus [15]. The variety of tumor types seen in these cases and in the current case suggests that mixed tumors such as these arise from a stem cell that is capable of differentiating in multiple directions. In the current case, each tumor morphology was positive for p53, which suggests that a p53 mutation could have been an early genetic event in the tumor stem cell. Due to the scarcity of cases, the prognosis of mixed tumors with several different morphologies is uncertain but likely poor.

In conclusion, this is the first report of a mixed large cell neuroendocrine carcinoma and adenocarcinoma with spindle cell and clear cell features in the extrahepatic bile duct. The tumor was highly aggressive, and the patient died with recurrent and metastatic disease five months after surgery.

## Figures and Tables

**Table 1:** Immunohistochemical results for each tumor morphology.

Morphology	AE1/3	CK7	Vim	NE	CA19-9	AFP	Hep	CD117	P53	Ki67
Adeno	++	++	−	+	+	Focal	Focal	−	+	50%
Clear cell	+	+	−	Scattered single cells	+	Focal	+	−	+	20%
LCNEC	+	++	−	+	−	−	Focal	+	+	80%
Spindle	Focal	Focal	+	Focal	Focal	−	−	−	+	50%

Adeno: adenocarcinoma; LCNEC: large cell neuroendocrine carcinoma; spindle: spindle cell carcinoma; AE1/3: cytokeratin cocktail AE1/AE3; CK7: cytokeratin 7; Vim: vimentin; NE: neuroendocrine markers chromogranin, synaptophysin, and CD56; AFP: alpha fetoprotein; Hep: Hep Par 1; LCNEC: large cell neuroendocrine carcinoma.
